# Supplementary material for: Insights into Early Recovery from Influenza Pneumonia by Spatial and Temporal Quantification of Putative Lung Regenerating Cells and by Lung Proteomics
Source: Cells. 2019 Aug 26;8(9):975. doi: 10.3390/cells8090975 (PMC6769472; doi:10.3390/cells8090975)
Supplement: Supplementary file 1 [file cells-08-00975-s001.pdf]

Supplementary Figures

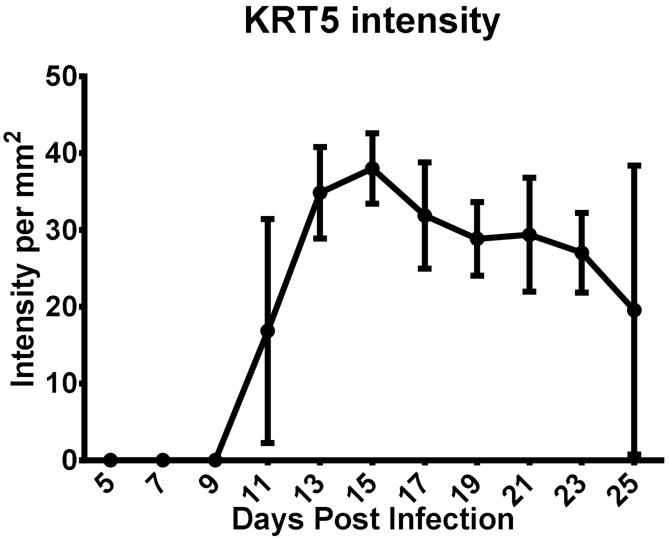

Supplementary Figure S1. Trend of KRT5 intensity per unit area of KRT5-positive pods in mouse infected lungs over time. Quantified by ImageJ software, the brightness intensity of KRT5 staining (in units per mm^2) increased from 9 dpi, reached a peak at 15 dpi, before decreasing until 25 dpi. At 25 dpi, the relatively large error bar was attributed to the absence of DASCs in one mouse. KRT5 expression was not detected in uninfected mouse lungs.

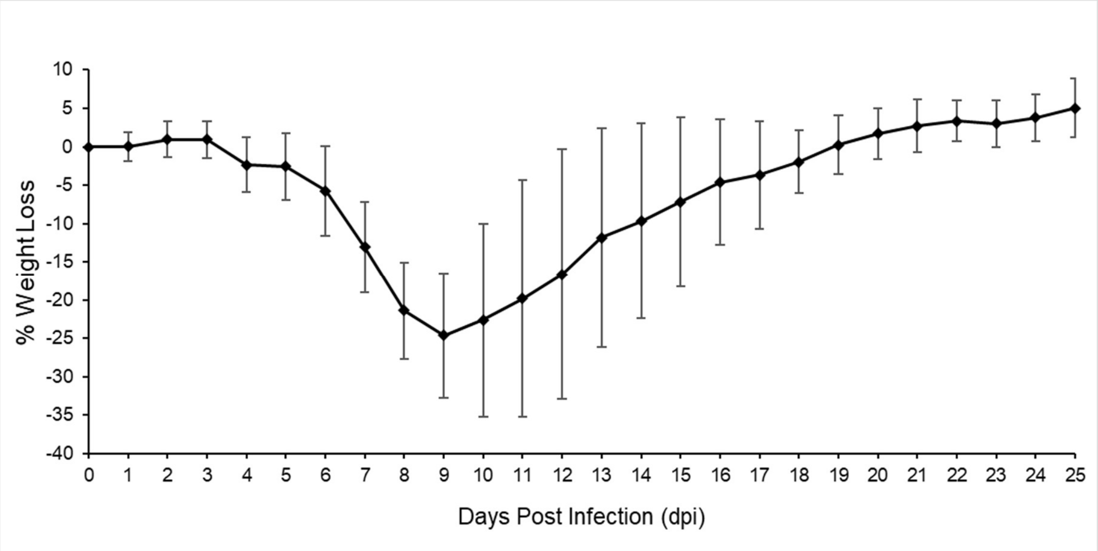

Supplementary Figure S2. Percentage weight loss of mice infected with a sub-lethal dose of 20 PFU of influenza virus H1N1 PR8. Upon intra-tracheal infection at day 0, the mice began to lose weight significantly from 5 dpi onwards, reaching peak weight loss of 25% at 9 dpi, before recovering and regaining their original weight by 20 dpi. The body weights of control uninfected mice were monitored until 7 dpi, but they did not exhibit any weight loss.

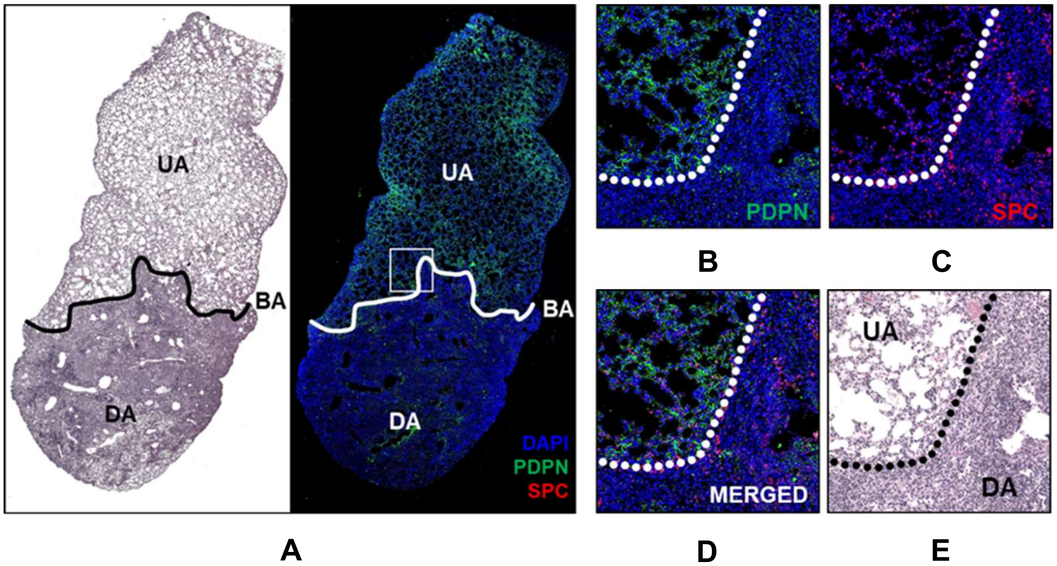

Supplementary Figure S3. Representative demarcation of the damaged area (DA), undamaged area (UA), and boundary area (BA) of a typical infected mouse lung at 13 dpi. In the H&E section of infected lung, the DA is characterized by the loss of the normal alveolar architecture with negligible expression of PDPN, a marker of alveolar type I cells **(A)**. Representative images of the boundary area between DA and UA at higher magnification of the area bounded by the white square **(B – E)**.

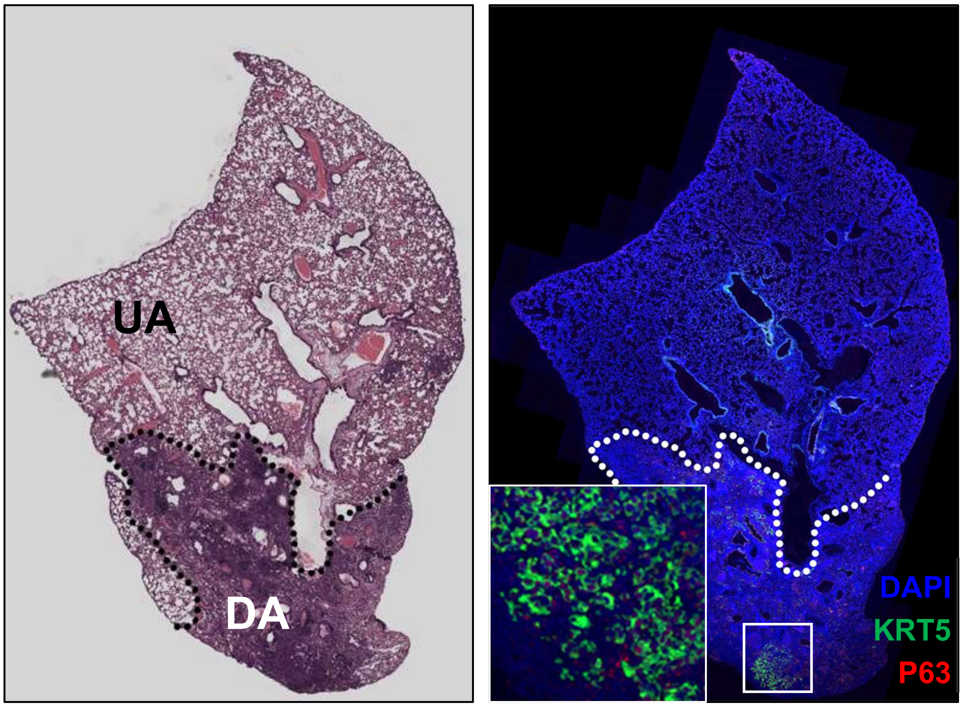

Supplementary Figure S4. DASCs are found only in the damaged area of infected lungs. We demarcated the infected mouse lungs as exemplified in this representative section at 13 dpi. DASCs with positive staining for KRT5 (and/or P63) were observed exclusively in the damaged area, but not in the undamaged area. No KRT5-positive expression was detected

in the lungs of control uninfected mice (data not shown). Bottom left inset of right image depicts higher magnification of the area within the white square.

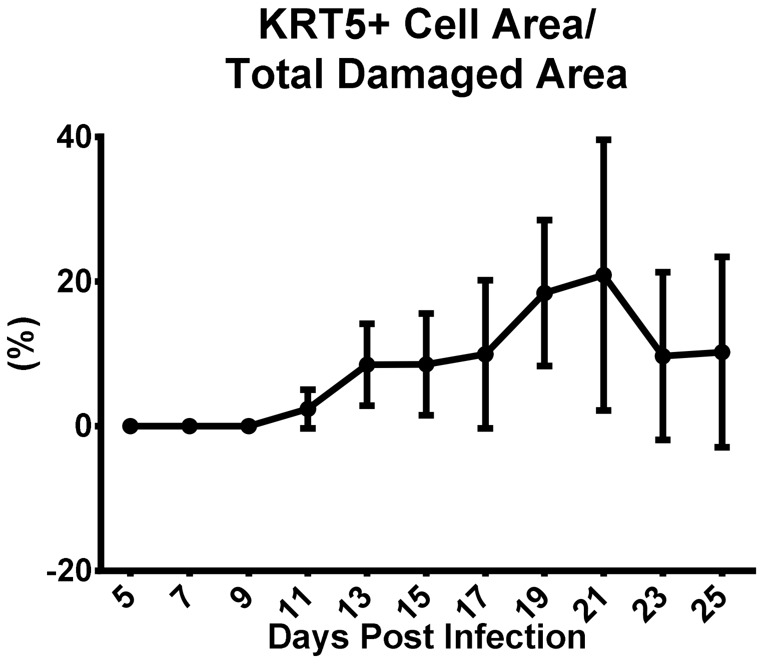

Supplementary Figure S5. Proportion of DASCs in the total damaged area over time. KRT5-positive DASCs began to appear at 11 dpi, attaining a maximum of 21% of the total damaged area at 21 dpi, before decreasing until 25 dpi.

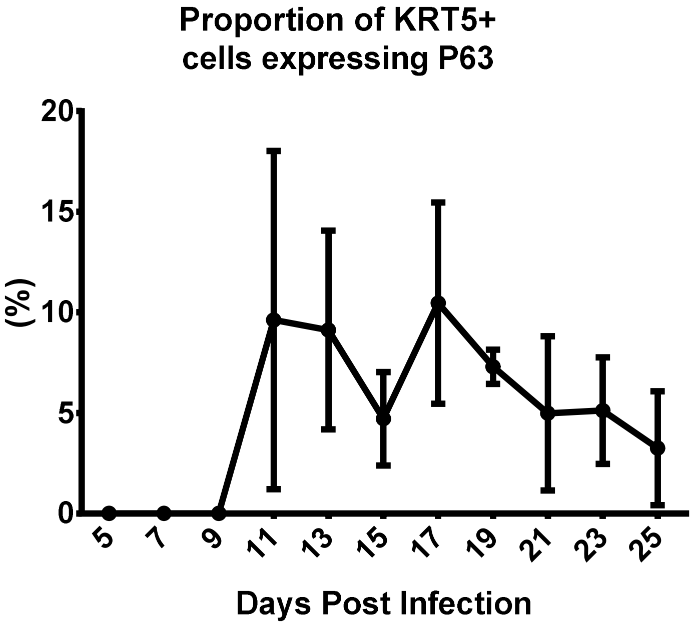

Supplementary Figure S6. Only a small fraction of KRT5-positive cells also co-express P63. We observed that only an average of 3–10% of the KRT5-positive cells also co-expressed the P63 marker.

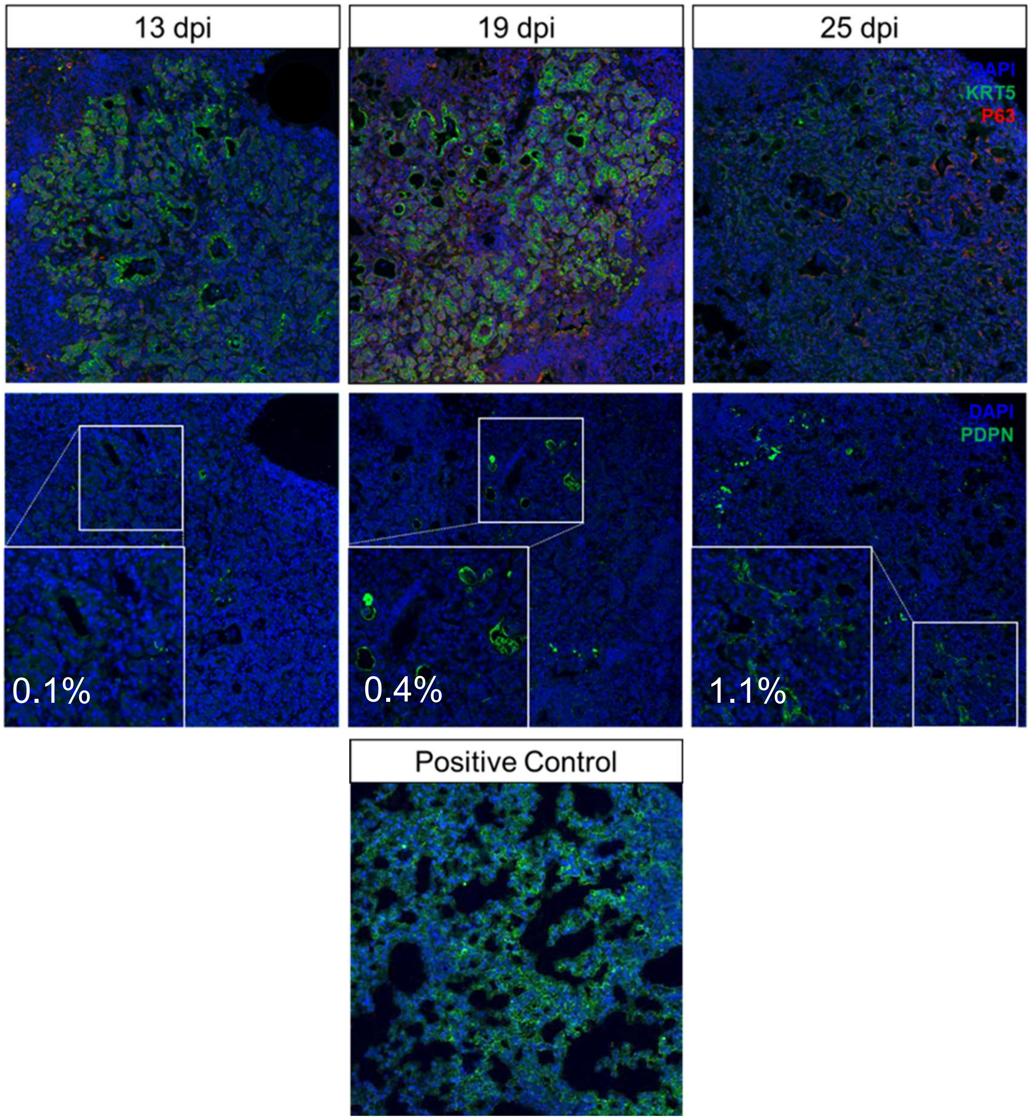

Supplementary Figure S7. About 1% or less of DASCs express the alveolar type I epithelial cell marker PDPN up to 25 dpi. For correlation with KRT5 and P63 staining of DASCs, the same lung sections in Figure 2 were subjected to staining for PDPN. Patchy PDPN staining was observed in lumens within the DASCs at 19 and 25 dpi. Notably, only about 1% or less of DASCs expressed PDPN. The positive control was represented by another undamaged area of the same lung section at 19 dpi, and showed widespread and strong PDPN staining as expected.

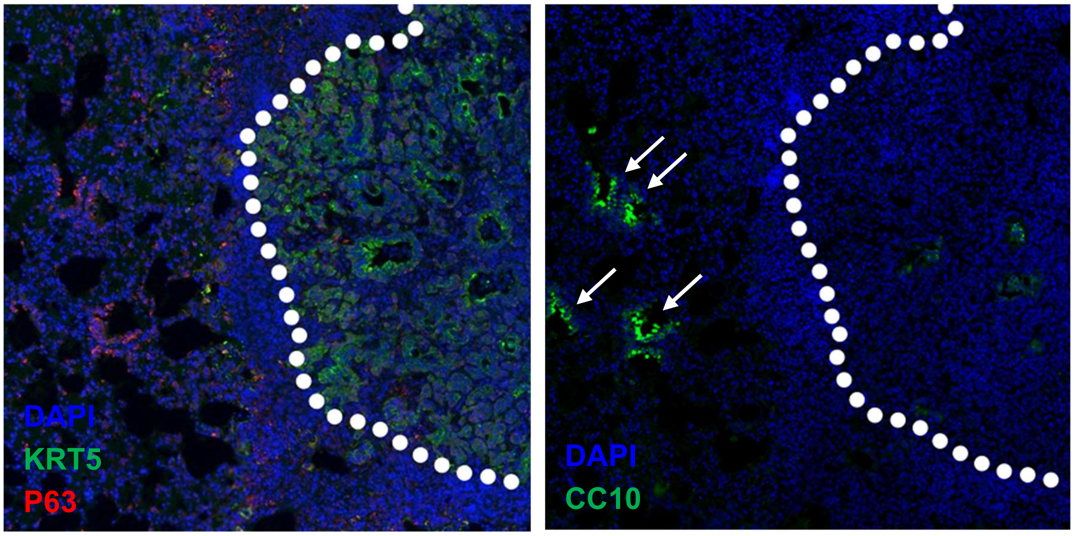

Supplementary Figure S8. DASCs do not express the Club cell marker CC10. No expression of CC10 was detected in the DASCs at 13, 19 and 25 dpi. The representative image at 13 dpi shows little to no CC10 staining in the area containing KRT5-positive DASC pods (which is encircled by the white dotted line). However, CC10 staining was detected in the lung area outside of the DASC pods (arrows).

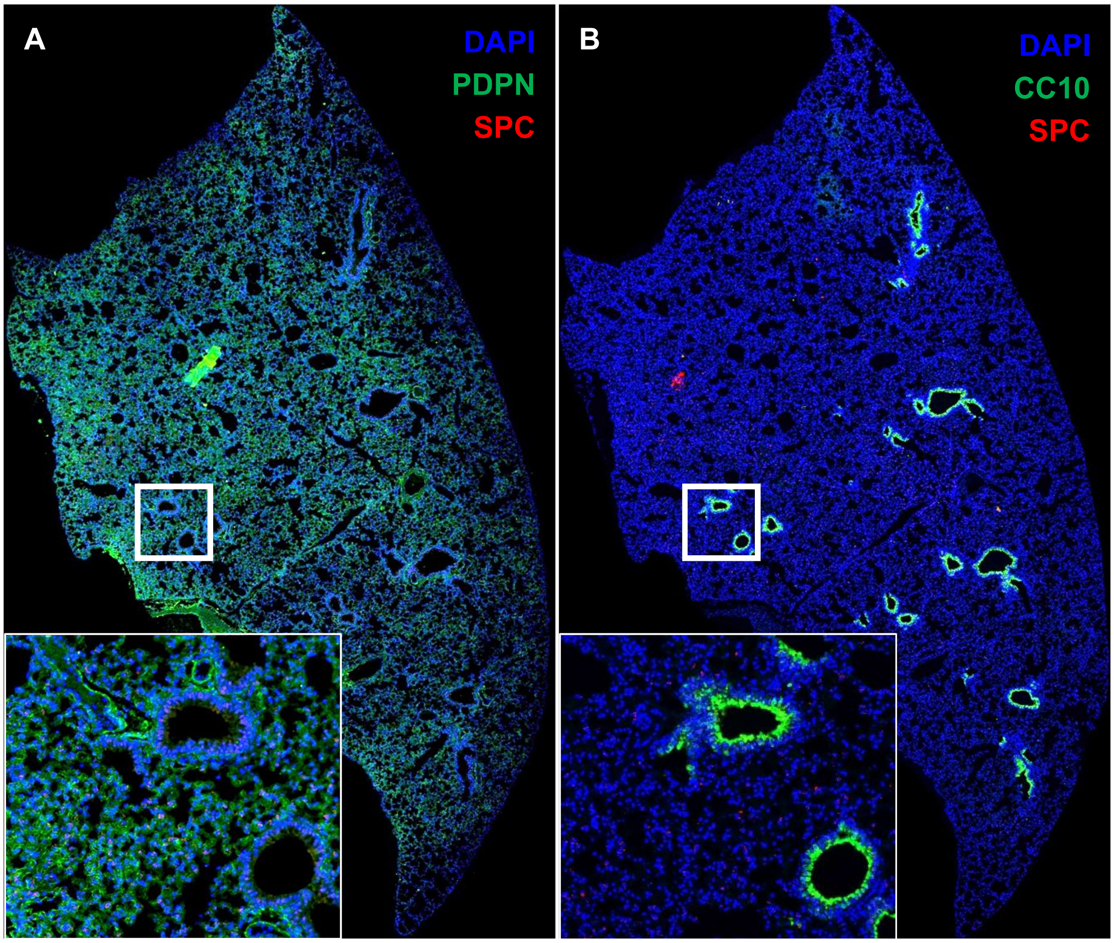

Supplementary Figure S9. PDPN and CC10 are widely expressed in lungs of control mice subjected to mock infection with PBS. To authenticate the immunofluorescence staining for PDPN and CC10, lungs of uninfected mice were stained to detect these markers. As expected, there was widespread PDPN expression in the uninfected lungs **(A)**. CC10 staining was localized in the bronchioles of uninfected lungs, where club cells reside **(B)**. Bottom left insets depict higher magnification of the areas within the white squares.

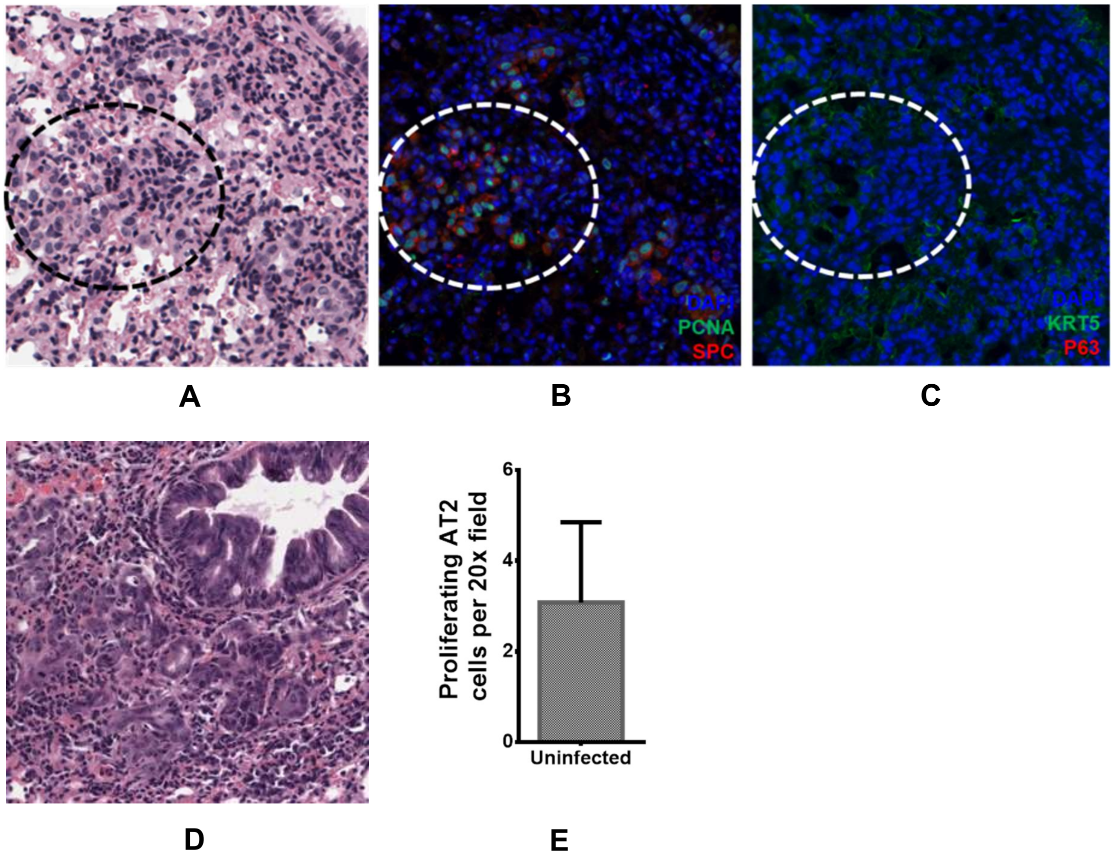

Supplementary Figure S10. The distribution and organization of proliferating AT2 cells are distinct from DASCs. Based on their morphology by H&E staining, proliferating AT2 cells in infected lungs **(circled in A, B, C)** displayed enlarged and uncondensed nuclei similar to DASCs in pods **(D)**, but expressed very low levels of KRT5 with absent P63 expression **(C)**. However, DASCs differed by their pod-like organization as well as their close proximity to bronchioles **(D)**. Hence, proliferating AT2 cells are likely to be a distinct cell population from DASCs. In control uninfected lungs at 7 days after mock infection, only a small number of these proliferating AT2 cells were detected **(E)**.

Supplementary Table S1. The top 15 processes up-regulated at 7 dpi based on protein functions by Gene Ontology (GO). Immune-related processes are highlighted in **bold font**. Statistically significant hypergeometric P values of less than 0.05 are shown in the last column.

GO term	Function	P value
GO:0072562	blood microparticle	0
GO:0005615	extracellular space	0
GO:0070062	extracellular exosome	8.88E-16
GO:0002376	**immune system process**	1.91E-14
GO:0010466	negative regulation of peptidase activity	1.07E-11
GO:0045087	**innate immune response**	1.91E-09
GO:0007596	blood coagulation	1.06E-08
GO:0006953	**acute-phase response**	4.70E-08
GO:0007599	hemostasis	9.21E-08
GO:0010951	negative regulation of endopeptidase activity	6.65E-07
GO:0051607	**defense response to virus**	1.88E-06
GO:0006412	translation	2.75E-06
GO:0030117	membrane coat	4.08E-05
GO:0071346	**cellular response to interferon-gamma**	4.97E-05
GO:0043434	response to peptide hormone	6.80E-05

Supplementary Table S2. The top 15 processes down-regulated at 7 dpi, with processes associated with cell adhesion in **bold font**.

GO term	Function	P value
GO:0005925	**focal adhesion**	3.33E-16
GO:0070062	extracellular exosome	2.55E-15
GO:0005739	mitochondrion	4.00E-15
GO:0043209	myelin sheath	1.19E-14
GO:0007155	**cell adhesion**	4.33E-14
GO:0031012	extracellular matrix	1.64E-10
GO:0055114	oxidation-reduction process	3.47E-10
GO:0005856	cytoskeleton	2.42E-09
GO:0042383	sarcolemma	1.01E-08
GO:0008152	metabolic process	1.02E-08
GO:0005913	**cell-cell adherens junction**	1.64E-08
GO:0005578	proteinaceous extracellular matrix	6.33E-08
GO:0001725	stress fiber	7.91E-08
GO:0007160	**cell-matrix adhesion**	1.23E-07
GO:0030054	**cell junction**	1.99E-07

Supplementary Table S3. The top 15 processes up-regulated at 15 dpi, with immune-related processes in **bold font**.

GO term	Function	P value
GO:0072562	blood microparticle	0
GO:0070062	extracellular exosome	0
GO:0006412	translation	6.15E-14
GO:0005615	extracellular space	3.66E-13
GO:0002376	**immune system process**	1.29E-09
GO:0006413	translational initiation	4.48E-08
GO:0007596	blood coagulation	1.20E-07
GO:0005737	cytoplasm	1.71E-07
GO:0007599	hemostasis	8.87E-07
GO:0010466	negative regulation of peptidase activity	2.82E-06
GO:0045087	**innate immune response**	4.35E-06
GO:0071346	**cellular response to interferon-gamma**	5.13E-06
GO:0000502	proteasome complex	8.05E-06
GO:0005783	endoplasmic reticulum	2.29E-05
GO:0009986	cell surface	2.30E-05

Supplementary Table S4. The top 15 processes down-regulated at 15 dpi, with processes associated with cell adhesion in **bold font**.

GO term	Function	P value
GO:0005739	mitochondrion	0
GO:0043209	myelin sheath	8.88E-16
GO:0005925	**focal adhesion**	1.44E-15
GO:0055114	oxidation-reduction process	1.44E-14
GO:0006099	tricarboxylic acid cycle	9.65E-14
GO:0070062	extracellular exosome	2.14E-12
GO:0007155	**cell adhesion**	9.54E-12
GO:0008152	metabolic process	1.62E-10
GO:0005913	**cell-cell adherens junction**	1.13E-07
GO:0005743	mitochondrial inner membrane	2.61E-07
GO:0005856	cytoskeleton	4.19E-07
GO:0043234	protein complex	4.99E-07
GO:0031012	extracellular matrix	5.26E-07
GO:0005759	mitochondrial matrix	3.37E-06
GO:0030054	**cell junction**	3.83E-06
